# Effect of sesamol on damaged peripheral nerves: Evaluation of functional, histological, molecular, and oxidative stress parameters

**DOI:** 10.22038/AJP.2022.20663

**Published:** 2022

**Authors:** Nastaran Amerian, Athar Talebi, Manouchehr Safari, Hamid Reza Sameni, Ali Ghanbari, Parisa Hayat, Moslem Mohammadi, Maryam Ardekanian, Sam Zarbakhsh

**Affiliations:** 1 *Nervous System Stem Cells Research Center, Semnan University of Medical Sciences, Semnan, Iran*; 2 *Research Center of Physiology, Semnan University of Medical Sciences, Semnan, Iran*; 3 *Cellular and Molecular Research Center, Iran University of Medical Sciences, Tehran, Iran*; 4 *Department of Physiology, Molecular and Cell Biology Research Center, Faculty of Medicine, Mazandaran University of Medical Sciences, Sari, Iran*; 5 *Department of Biotechnology, Faculty of Medicine, * *Semnan University of Medical Sciences, Semnan, Iran*

**Keywords:** Nerve growth factor, MPZ protein, Oxidative stress, Peripheral nerve injuries, Regeneration, Sesamol

## Abstract

**Objective:**

Peripheral nerve injury is a clinical problem that may cause sensory and motor inabilities. Sesamol is an antioxidant that can help in repairing damaged central nervous system (CNS) and other organs. The present study aimed to investigate whether the antioxidant effects of sesamol could improve the function, structure, and myelination in rats’ damaged peripheral nervous system (PNS).

**Materials and Methods:**

In this study, 28 adult male Wistar rats were randomly divided into four groups. In the sham group, the sciatic nerve was exposed and restored to its place without inducing crush injury. The control received DMSO (solvent) and the two experimental groups received 50 or 100 mg/kg sesamol intraperitoneally for 28 days after sciatic nerve crush injury, respectively. Next, sciatic function index (SFI), superoxide dismutase (SOD) activity, malondialdehyde (MDA) level, expression of nerve growth factor (NGF) and myelin protein zero (MPZ) proteins in the sciatic nerve, and histological indices of the sciatic nerve and gastrocnemius muscle were evaluated.

**Results:**

The results showed that sesamol reduced oxidative stress parameters, increased expression of NGF and MPZ proteins, and improved function and regeneration of the damaged sciatic nerve. Furthermore, a significant regeneration was observed in the gastrocnemius muscle after treatment with sesamol. Although administration of both doses of sesamol was useful, the 100 mg/kg dose was more effective than the 50 mg/kg one.

**Conclusion:**

The results suggest that sesamol may be effective in improving damaged peripheral nerves in rats by reducing oxidative stress and increasing the expression of NGF and MPZ proteins.

## Introduction

Peripheral nerve damage is a clinical problem that may cause complications such as atrophy and irreversible muscle fibrosis and can limit a person’s activity by causing sensory and motor disabilities (Zhou et al., 2020 ▶). When peripheral nerves are damaged, the repairing process begins while the regenerating time is limited and if it takes too long, recovery will not take place (Paskal et al., 2020 ▶).

Antioxidants are substances that protect cells against harmful free radicals (Sameni et al., 2018 ▶; Zarbakhsh et al., 2019 ▶; Zarbakhsh, 2021 ▶). Some antioxidants have the potential to accelerate the growth of axons (Hsiang et al., 2011 ▶; Zhao et al., 2017 ▶; Kurt Oktay et al., 2021 ▶) and improve the repairing process of damaged peripheral nerves (Renno et al., 2017 ▶; Wang et al., 2011 ▶; Hajimoradi et al., 2015 ▶). In this regard, it has been shown that some antioxidants can also protect the peripheral nerves in diabetic rats (Farshid and Tamaddonfard, 2015 ▶). In addition, aloe vera as an antioxidant showed neuroprotective effects versus ischemia/reperfusion injury in rat sciatic nerve (Guven et al., 2016 ▶). Sesamol (3, 4-methylenedioxy phenol) is a natural organic compound of sesame seeds and sesame oil with antioxidant properties and potential therapeutic benefits (Geetha et al., 2009 ▶). Antioxidant, protective, and restorative effects of sesamol have been demonstrated in some tissues and organs (Bosebabu et al., 2020 ▶). Moreover, sesamol can elevate the level of nerve growth factor (NGF) in the brain, which promotes the growth of axons in the central nervous system (CNS) (Hassanzadeh and Hassanzadeh, 2013 ▶).

In peripheral nerve damage, the amount of NGF in Schwann cells increases rapidly (Shakhbazau et al., 2012 ▶). NGF promotes myelination, activates autophagy in Schwann cells to increase the clearance of myelin debris, and accelerates peripheral nerve regeneration (Li et al., 2020 ▶; Chan et al., 2004 ▶). In addition, Schwann cells produce myelin protein zero (MPZ) which is the most important protein for myelination in the peripheral nervous system (PNS) (da Silva et al., 2015 ▶).

Although some studies have investigated the restorative effects of sesamol on CNS disorders, the action of sesamol on PNS regeneration is unclear. In this study, we hypothesized that sesamol could promote the repair of damaged sciatic nerve. Therefore, in this study, for the first time, the effects of sesamol on the function, structure, and myelination of rat damaged peripheral nerves were evaluated.

## Materials and Methods

**Animals**

In this study, 28 adult male Wistar rats (250-300 g) were used. The rats were maintained in a controlled temperature (25±2°C) environment with free access to rodent diet and water. All experimental procedures were confirmed by the ethical committee of Semnan University of Medical Sciences (Semnan, Iran). The ethical approval number is IR.SEMUME.REC.1398.248.

**Creating crush injury model**

**Figure 1:**
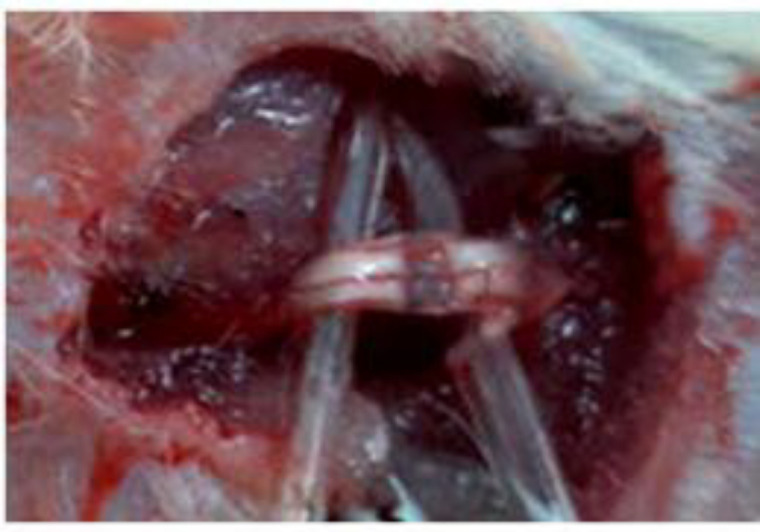
The site of crush injury in the sciatic nerve induced by clamping with the non-serrated hemostatic forceps. The nerve was cut while the epineurium sheath was preserved

To create the model of crush injury, the rats were anesthetized by intraperitoneal injection of 80 mg/kg ketamine and 10 mg/kg xylazine. The right sciatic nerve was exposed in the middle of the thigh and crush injury was performed by clamping the nerve with non-serrated hemostatic forceps for 30 sec, so that the connection between the proximal and distal axons was cut to a length of 3 mm while the nerve epineurium sheath was preserved (Figure 1). Then, the nerve was returned to its place and the skin was sutured with 4-0 silk (Abdolmaleki et al., 2020 ▶).

**Animal groups**

The rats were randomly divided into four groups (n=7 in each group). In the sham group, the sciatic nerve was exposed and then, restored to its place without inducing crush injury. In the control group, after creating the crush injury, 0.5% dimethyl sulfoxide (DMSO) (used as the sesamol solvent) (Sigma, Swiss) was intraperitoneally injected daily for 28 days. In the experimental groups, after creating the crush injury, 50 or 100 mg/kg of sesamol (Sigma, Germany) was intraperitoneally injected daily for 28 days (Moattari et al., 2018 ▶). Then, after examining the sciatic nerve function, the nerve and gastrocnemius muscle were removed to evaluate the parameters of oxidative stress, expression of NGF and MPZ proteins in the sciatic nerve, and histology of the sciatic nerve and gastrocnemius muscle. The length of sciatic nerve crush injury was 3 mm. To remove the nerve, 1 mm proximal to the injury site and 3 mm distal to the injury site (i.e. a total of 7 mm of the nerve) was removed. In each group, three sciatic nerves were used to measure oxidative stress parameters, and four sciatic nerves were divided into two equal parts and used for western blotting and histology evaluations.

**Functional evaluation**

The function of the sciatic nerve was calculated by the sciatic function index (SFI). First, the hind feet of rats were colored with ink and then, rats were allowed to walk in a tunnel, and footprints were recorded on papers placed on the floor of the tunnel. The SFI was calculated as follows: 

SFI=-38.3×(EPL-NPL)/NPL+109.5×(ETS-NTS)/NTS+13.3×(EITS-NITS)/NITS-8.8. 

EPL: experimental print length, NPL: normal print length, ETS: experimental toe spread, NTS: normal toe spread, EITS: experimental intermediary toe spread, and NITS: normal intermediary toe spread. Generally, the SFI for normal nerve function is near 0 and for total dysfunction is around -100 (Zarbakhsh et al., 2013 ▶).

**Preparation of the sciatic nerve homogenate**

The right sciatic nerve was rinsed with KCl (150 mM) to remove any red blood cells and clots. Then, the nerve was homogenized in 1 ml ice-cold KCl (150 mM). After centrifugation (20 min at 4,000 × g) at 4°C, the supernatant was collected and stored at −80°C for oxidative stress analyses. The total protein concentration of the tissue sample in the supernatant was defined by the Bradford method using bovine serum albumin (BSA) as the standard (Bradford, 1976 ▶).

**Measurement of superoxide dismutase (SOD) activity**

SOD activity in the sciatic nerve tissue was measured using an SOD assay kit (Teb Pazhouhan Razi, Tehran, Iran) according to the manufacturer’s instructions. A mixture of the supernatant, SOD enzyme solution, SOD assay buffer, and chromogenic reagent were incubated at 37°C for 20 min. Then, the absorbance was measured at 450 nm using a spectrophotometer (Pharmacia Biotech, Sweden). The SOD activity is expressed as units of enzyme/mg protein (U/mg protein).

**Measurement of malondialdehyde (MDA) level**

MDA level in the sciatic nerve tissue was measured by an MDA assay kit (Teb Pazhouhan Razi, Tehran, Iran) according to the manufacturer’s instructions. The color made by the reaction of sample MDA with thiobarbituric acid was measured at 532 nm using a spectrophotometer (Pharmacia Biotech, Sweden). The MDA level is expressed as nmol/mg protein.

**Western blot assay**

The expression of NGF and MPZ proteins in the sciatic nerve tissue was assessed by western blotting. Briefly, after the sciatic nerve was lysed with radio-immunoprecipitation assay (RIPA) buffer and protease inhibitor (Roche, Switzerland), and the mixture was centrifuged. The proteins were separated by a polyacrylamide gel electrophoresis (PAGE), then transferred onto nitrocellulose membranes (Amersham Biosciences, USA) and blocked with 5% skim milk. The membranes were then incubated with primary antibodies for NGF (1:1000, Abcam, USA), MPZ (1:1000, Abcam, USA), and β-actin (1:1000, Abcam, USA) overnight at 4°C. After three washes with 0.1% Tween 20 (TBS-T), the membranes were incubated with the secondary antibody, goat anti-rabbit conjugated with HRP (1:5000) (Santa Cruz Biotechnology, USA). Immunoreactive bands were demonstrated using a chemiluminescence detection kit (Amersham Biosciences, USA) and quantified by ImageJ software (da Silva et al., 2015 ▶).

**Histological assessment of the sciatic nerve**

To evaluate the morphology of the sciatic nerve, the nerve was fixed in 2.5% glutaraldehyde solution and then, in 2% osmium tetroxide. After embedding in Epon resin, semi-thin sections (700 nm) were obtained using an ultramicrotome and stained with toluidine blue. Finally, the histomorphometric analyses including nerve fiber area/µm^2^, nerve fiber diameter/µm, axon area/µm^2^, axonal diameter/µm, and myelin sheath thickness/µm (nerve fiber diameter - axonal diameter)/2 were calculated by ImageJ software (Hao et al., 2017 ▶; Renno et al., 2017 ▶).

**Histological assessment of the gastrocnemius muscle**

To evaluate the morphology of the gastrocnemius muscle, along with bringing out the right sciatic nerve, the right gastrocnemius muscle was retrieved. The muscle was fixed in 4% paraformaldehyde and after processing, sections with the thickness of 5 µm were prepared. Five sections were randomly selected from each muscle and stained with hematoxylin and eosin (H&E). Finally, histomorphometric analysis of the gastrocnemius muscle, including evaluation of cross-sectional muscle fiber area/mm^2^, an indicator of muscle atrophy, was performed by ImageJ software (La et al., 2019 ▶).

**Statistical analyses**

Data analyses were performed by one-way analysis of variance (ANOVA) followed by Tukey’s *post hoc* test. Data are presented as mean±S.E.M and a p<0.05 was considered statistically significant.

## Results

**Functional analysis**

**Figure 2:**
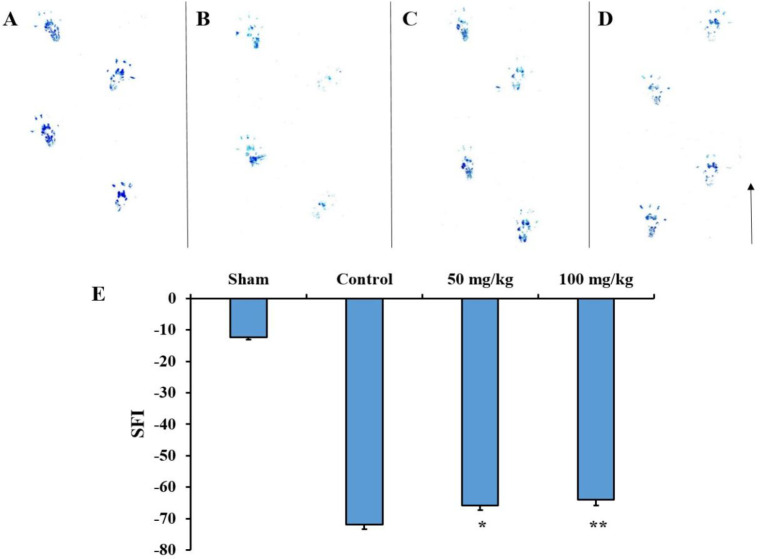
Effects of sesamol on the sciatic function index (SFI) four weeks after sciatic nerve injury. The footprint of rat hind feet in the sham (A), control (B), 50 mg/kg sesamol (C), and 100 mg/kg sesamol (D) groups. The results of the SFI values (E). The arrow shows the walking direction. *p<0.05 and **p<0.01 vs. the control group

Effects of sesamol on the SFI values were evaluated four weeks after sciatic nerve injury. To do that, the footprint of rats hind feet was collected and analyzed. The results showed that administration of 50 mg/kg (p<0.05) and 100 mg/kg (p<0.01) sesamol were significantly more effective in the recovery of sciatic nerve function than DMSO (i.e. the control group) (Figure 2).

**Evaluation of oxidative stress parameters**

**Figure 3:**
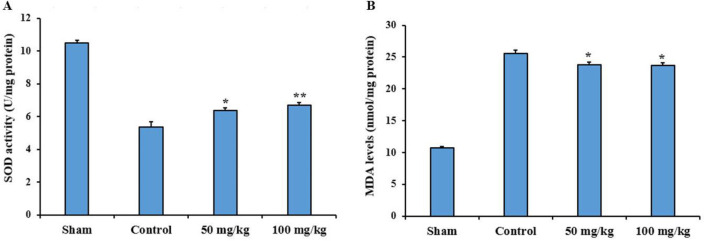
Effects of sesamol on superoxide dismutase (SOD) activity (A) and malondialdehyde (MDA) level (B) in the damaged sciatic nerves four weeks after surgery. *p<0.05 and **p<0.01 vs. the control group

The results of SOD and MDA assay showed that administration of 50 mg/kg and 100 mg/kg sesamol significantly increased SOD activity compared to the control group (p<0.05 and p<0.01, respectively). Administration of both doses of sesamol significantly decreased MDA levels compared to the control group (p<0.05) (Figure 3). 

**Evaluation of the expression of NGF and MPZ proteins**

**Figure 4:**
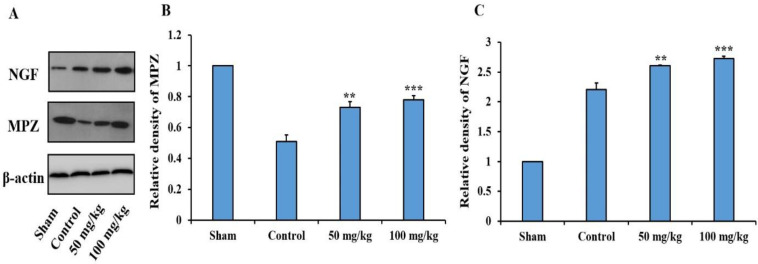
Effects of sesamol on the protein expression of nerve growth factor (NGF) and myelin protein zero (MPZ) in the damaged sciatic nerves four weeks after surgery. Protein levels were measured by western blotting and densities of protein bands were normalized against corresponding β-actin. Immunoblot of NGF, MPZ, and β-actin proteins (A) and relative densities of NGF (B) and MPZ (C) proteins. **p<0.01 and ***p<0.001 vs. the control group

The results of the effects of sesamol on the expression of NGF and MPZ proteins in the damaged sciatic nerves showed that administration of 50 mg/kg (p<0.01) and 100 mg/kg (p<0.001) sesamol significantly increased the expression of both proteins compared to the control group (Figure 4**).**

**Histological evaluation of the sciatic nerve**

**Figure 5:**
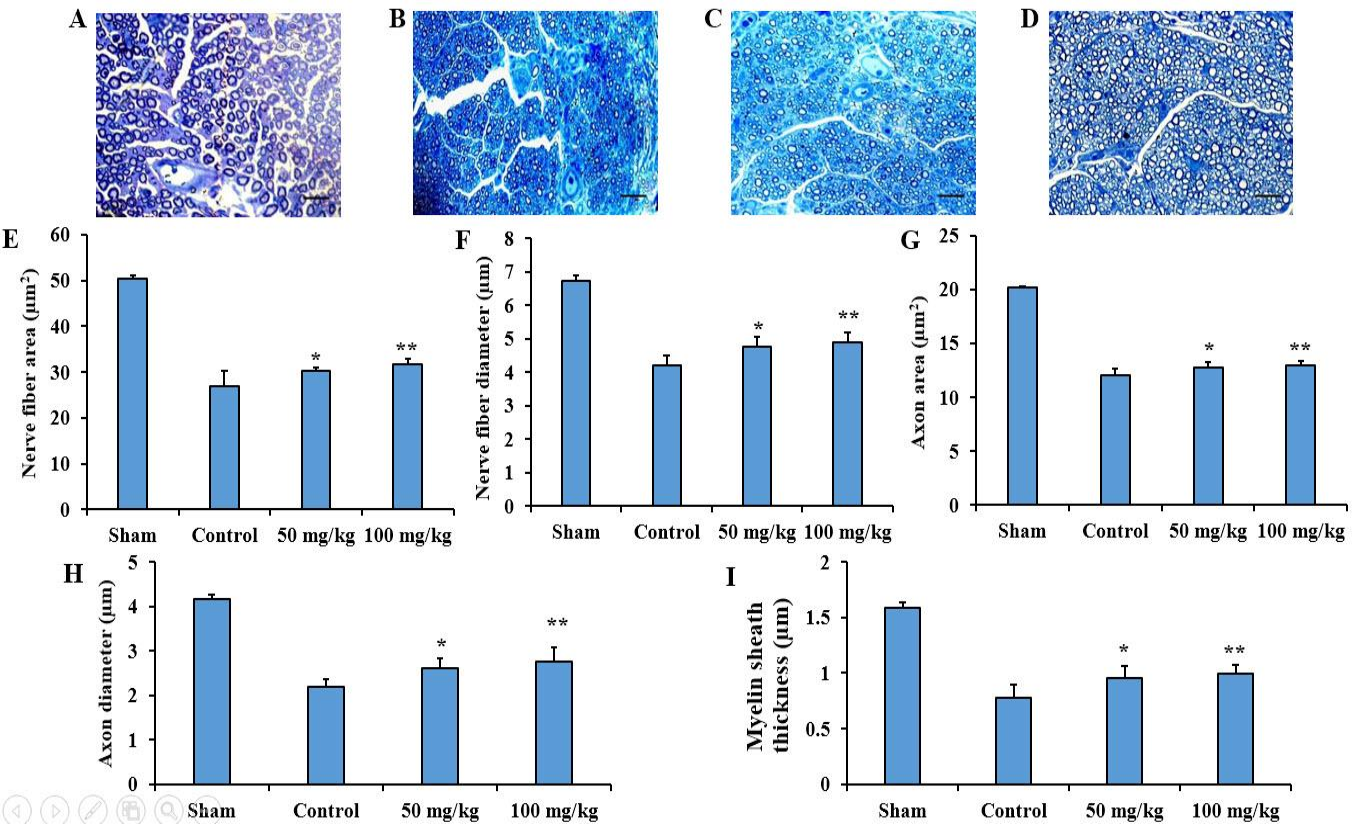
Effects of sesamol on histological parameters in the damaged sciatic nerves. Four weeks after surgery, semi-thin cross-sections from the sciatic nerves were stained with toluidine blue in the sham (A), control (B), 50 mg/kg sesamol (C), and 100 mg/kg sesamol (D) groups. The results are shown as nerve fiber area (E), nerve fiber diameter (F), axon area (G), axonal diameter (H), and myelin sheath thickness (I). Scale bars: 100 μm. *p<0.05 and **p<0.01 vs. the control group

The results of histological evaluation of the sciatic nerve in terms of the nerve fiber area, nerve fiber diameter, axon area, axonal diameter, and myelin sheath thickness showed that administration of 50 mg/kg (p<0.05) and 100 mg/kg (p<0.01) sesamol significantly improved the sciatic nerve regeneration compared to the control group (Figure 5). 

**Histological evaluation of the gastrocnemius muscle**

**Figure 6:**
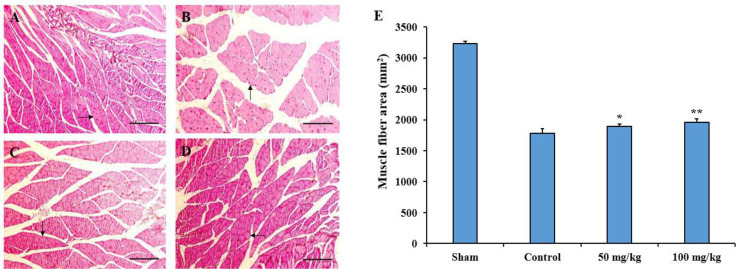
Effects of sesamol on histological parameters in the gastrocnemius muscle following sciatic nerve injury. Four weeks after sciatic nerve injury, cross-sections from the gastrocnemius muscles were stained with hematoxylin and eosin (H&E) in the sham (A), control (B), 50 mg/kg sesamol (C), and 100 mg/kg sesamol (D) groups. The results are shown as muscle fiber area (E). Scale bars: 100 µm. Arrows indicate muscle fibers. *p<0.05 and **p<0.01 vs. the control group

The results of histological evaluation of the gastrocnemius muscle in terms of the cross-sectional muscle fiber area demonstrated that administration of 50 mg/kg (p<0.05) and 100 mg/kg (p<0.01) sesamol significantly improved the muscle regeneration compared to the control group (Figure 6).

## Discussion

Following peripheral nerve injury, administration of neuroprotective drugs may be effective in enhancing axonal regeneration (Abdolmaleki et al., 2020 ▶). In the present study, the antioxidant effects of sesamol on the function, histological and myelination of rat damaged peripheral nerves were investigated. Overall, the results showed that sesamol improved the repair process of the damaged sciatic nerve, and administration of 100 mg/kg of sesamol was more effective than 50 mg/kg. These results were consistent with other findings of sesamol as a protective agent in a variety of neurodegenerative diseases in the CNS such as ischemic stroke, Parkinson’s disease, blood-brain barrier dysfunction, memory impairment, and emotional and cognitive derangements (Hong et al., 2015 ▶; Sonia Angeline et al., 2013 ▶; VanGilder and Huber, 2014 ▶; Liu et al., 2017 ▶; Kakkar et al., 2011 ▶). Moreover, the results of this study were consistent with those reported for other antioxidants such as curcumin, puerarin, resveratrol, and quercetin in improving the repair process of peripheral nerve injuries (Hsiang et al., 2011 ▶; Zhao et al., 2017 ▶; Zhang et al., 2020 ▶; Turedi et al., 2018 ▶). 

In the current study, sciatic nerve crush injury was applied as a standard model to evaluate peripheral nerve regeneration. Then, doses of 50 and 100 mg/kg of sesamol were intraperitoneally injected for 28 days. We selected these doses because other studies have confirmed their effectiveness (Hassanzadeh and Hassanzadeh, 2013 ▶; Vennila and Pugalendi, 2010 ▶).

The antioxidant effects of sesamol were evaluated by examining the SOD activity as an antioxidant parameter and MDA level as an oxidant parameter in the sciatic nerve. Administration of sesamol increased SOD activity and decreased MDA level. These results were consistent with other related studies. Ren et al. have reported that sesamol protected the mouse nervous system by reducing inflammation and MDA levels and increasing SOD activity in the serum and hippocampus (Ren et al., 2020 ▶). Zhang et al. showed that sesamol had neuroprotective properties by reducing MDA levels and increasing SOD activity in the rat hippocampus (Zhang et al., 2021 ▶). Gao et al. demonstrated that sesamol decreased oxidative stress in the injury of focal cerebral ischemia/reperfusion by decreasing MDA level and increasing SOD activity in the brain (Gao et al., 2017 ▶). 

To assess the function of the sciatic nerve, SFI was measured. SFI is a standard formula to assay sciatic nerve function in mice or rats (Zarbakhsh et al., 2013 ▶). Based on the results of histology and MPZ expression, sesamol has probably accelerated motor recovery by enhancing axonal myelination. The results of SFI were in agreement with another study done on mice. Hsu et al. have shown that sesame oil improves functional recovery in mouse sciatic nerve after 6 days by decreasing oxidative stress and increasing Nrf2 and GAP43 expression (Hsu et al., 2016 ▶).

In the histological evaluations, in addition to examining the morphology of the sciatic nerve, the morphology of the gastrocnemius muscle was examined because the morphology of gastrocnemius muscle is one of the indicators of repair of the damaged sciatic nerve. The more effective the treatment of damaged sciatic nerve, the less gastrocnemius muscle atrophy (Zarbakhsh et al., 2016 ▶). According to the results of histology, oxidative stress, and western blot, sesamol has probably promoted regeneration of the sciatic nerve and gastrocnemius muscle by decreasing oxidative stress and increasing the expression of NGF and MPZ proteins in the sciatic nerve. In this regard, Ren et al. have reported that sesamol regulates the Nrf2/Keap1 pathway which is a key pathway for antioxidants to remove reactive oxygen species (ROS) and other detrimental free radicals to protect cells from apoptosis and oxidative stress which shows that sesamol can be a potential neuroprotective agent (Ren et al., 2018 ▶).

Evaluation of the expression of MPZ and NGF proteins in the sciatic nerve was performed by western blotting. MPZ is a member of the immunoglobulin supergene family which is expressed by Schwann cells and is the most important protein expressed in the PNS myelin sheath. The more MPZ is expressed, the more myelin sheath formation and nerve repair occur (Shy, 2006 ▶). On the other hand, NGF is the first neurotrophic factor that stimulates the survival of neurons and the growth of axons (Li et al., 2020). Normally, the expression of NGF in the sciatic nerve is low. When the nerve is damaged, the amount of NGF expression suddenly increases. There is a direct relationship between increased expression of NGF and MPZ proteins with the improvement of damaged peripheral nerves (da Silva et al., 2015 ▶). The results showed that sesamol has the ability to increase the expression of these proteins in the damaged sciatic nerve, which increased myelination, axonal growth, and nerve repair. Regarding the NGF expression, Hassanzadeh and Hassanzadeh demonstrated that administration of 100 mg/kg sesamol increased NGF expression in the rat brain (Hassanzadeh and Hassanzadeh, 2013 ▶) which was consistent with our results. 

In the PNS, Schwann cells play an essential role in regulating the expression of MPZ and NGF proteins (da Silva et al., 2015 ▶). Lv et al. have reported that after an acute peripheral nerve injury, transcription of Nrf2 as an intrinsic antioxidant system is temporarily inactivated, so Schwann cell plasticity is weakened (Lv et al., 2018 ▶). Therefore, the use of external antioxidants in these conditions may help to regulate endogenous antioxidant pathways and improve Schwann cell function to repair damaged peripheral nerves. Nayak et al. have shown that sesamol can regulate endogenous antioxidant enzyme levels such as SOD, catalase, glutathione, and glutathione peroxidase (Nayak et al., 2013 ▶). Also, several studies have demonstrated that some antioxidants proliferate Schwann cells with different mechanisms, thereby improving the repairing process of damaged peripheral nerves. Zhu et al. have shown that ferulic acid as an antioxidant with neuroprotective properties can increase the proliferation and differentiation of Schwann cells through the MEK1/ERK1/2 signaling pathway, which increases remyelination in damaged peripheral nerves (Zhu et al., 2016 ▶). Another antioxidant, melatonin, promotes the proliferation and migration of Schwann cells through the SHH signaling pathway and inhibits apoptosis in Schwann cells by decreasing oxidative stress and mitochondrial dysfunction and upregulating Bcl-2, Wnt, and NF-kB signaling pathways (Pan et al., 2021 ▶; Tiong et al., 2019 ▶). In this regard, the antioxidant effects of sesamol on Schwann cell proliferation and function need further investigations. 

There was a limitation in this study. The number of samples was small, so more samples are needed and molecular mechanisms of sesamol require further investigations.

The results suggest that sesamol has neuroprotective effects and may be effective in improving the process of peripheral nerve repair in rats by reducing oxidative stress and increasing the expression of NGF and MPZ proteins. However, more research is required to confirm these findings.

## Conflicts of interest

The authors have declared that there is no conflict of interest.
